# Echocardiographic and Angiographic Assessment of Right Ventricular Function and Right Coronary Artery Stenosis in Acute Inferior Wall Myocardial Infarction

**DOI:** 10.7759/cureus.46403

**Published:** 2023-10-03

**Authors:** Rajneesh Kumar, Prakash Kumar, Prabin K Srivastava, Prashant Kumar

**Affiliations:** 1 Department of Cardiology, Rajendra Institute of Medical Sciences, Ranchi, IND

**Keywords:** right coronary artery stenosis, right ventricular function risk stratification, myocardial infarction, echocardiography, cardiovascular diseases, acute coronary syndrome

## Abstract

Background: Cardiovascular diseases (CVDs) are a global concern. CVD remains a primary cause of death despite reduced coronary heart disease death rates. Acute coronary syndrome (ACS) involves myocardial infarction (MI) and unstable angina, sharing mechanisms such as plaque instability. Our study assesses the right ventricular (RV) function's predictive value in acute inferior wall MI (IWMI) to identify high-risk patients with an elevated likelihood of experiencing severe cardiac complications, hemodynamic instability, or a higher mortality risk following an acute IWMI.

Methodology: The research was conducted in the Department of Cardiology at the Rajendra Institute of Medical Sciences (RIMS), Ranchi, from July 2021 to June 2022, following the necessary ethical approval. A cohort of 140 patients with IWMI, carefully chosen according to rigorous criteria, clearly understood the study's objectives before providing informed consent. The evaluations were conducted in the following order: clinical assessments, followed by blood testing, then echocardiography, and finally, coronary angiography. Furthermore, the study examined risk factors and utilized statistical methods to elucidate the associations between qualities and results.

Results: The study included 140 participants, with 61% being male and 39% female. Among the participants, 14% were aged 30-45, 50% were aged 46-60, and 30% were over 60. Age shows significant proportions in different categories. Diabetes, dyslipidemia, hypertension, and smoking/tobacco addiction did not differ among stenosis groups. Proximal right coronary artery (RCA) stenosis patients had elevated jugular venous pressure (JVP). The echocardiograms were performed within 48 hours of post-percutaneous coronary intervention, and significant differences between groups were observed. Participants with proximal stenosis had lower tricuspid annular plane systolic excursion (TAPSE) and right ventricular fractional area change (RVFAC), which showed compromised RV systolic function. Proximal stenosis patients had reduced systolic motion velocity (Sm), indicating impaired myocardial contraction. Echocardiographic parameters such as early diastolic velocity (Em), atrial contraction velocity (Am), Em/Am ratio (a marker of diastolic function), isovolumic relaxation time (IVRT), isovolumic contraction time (IVCT), and ejection time (ET) between groups were different, indicating distinct cardiac functions. Proximal stenosis increased the myocardial performance index (MPI), indicating cardiac impairment. The left ventricular ejection fraction (LVEF) was comparable in the two stenosis groups, indicating similar left ventricular performance.

Conclusion: Echocardiography showed significant RV function differences in acute inferior wall ST-segment elevation myocardial infarction (STEMI) patients with proximal and distal RCA lesions. RV dysfunction is linked to right ventricle myocardial infarction (RVMI), and echocardiographic markers can provide valuable insights. Results emphasize that acute inferior wall STEMI is diagnosed by electrocardiogram (ECG) criteria, particularly ST-segment elevation. However, these markers emphasize the importance of RV assessment in RCA involvement assessment. These findings suggest that RV function can help diagnose acute inferior wall STEMI RCA involvement. In acute inferior STEMIs, RV function echocardiography is essential for RCA lesion location.

## Introduction

Cardiovascular disease (CVD) is a significant problem in developed countries and is growing in developing countries [1]. Over the past 40 years, the mortality rate from coronary heart disease has dropped by two-thirds in the United States. This decline is the result of improved identification and management of risk factors as well as advances in treating CVD, heart failure, and arrhythmias. Despite these advancements, CVD remains the leading cause of mortality and morbidity in developed nations. This determination highlights the significance of enhancing public health outcomes and addressing complex health issues. Acute coronary syndrome (ACS) can result in numerous cardiac disorders. Myocardial infarction (MI) and unstable angina can occur with or without ST-segment elevation. These conditions are brought on by unstable coronary plaques, abnormal blood clotting, and constriction of the blood vessels. This results in subendocardial or transmural ischemia, characterized by an insufficient supply of blood to the myocardium [2].

MIs of the left ventricle (LV) have received considerable attention, but the effects of the right ventricle (RV) MIs on patients were not fully understood until 1974. In clinical practice, the heart's right ventricle (RV) is almost always supplied with blood by the right coronary artery (RCA), regardless of coronary artery dominance [3]. This generally means that MIs in this region are less severe [4]. A significant portion of the myocardium, which comprises cardiac muscle tissue, can survive without immediate medical care. It is important to clarify that myocardial survival does not solely pertain to left ventricular function; instead, it encompasses the entire heart muscle responsible for its pumping action [5]. In the context of cardiovascular physiology, it is observed that most of the population, exceeding 80%, exhibits a right-sided dominant cardiovascular system. In such cases, the right coronary artery (RCA) supplies the right ventricle (RV) with blood. However, it is important to note that even in individuals with a left-dominant system, the RCA may and often does contribute to the blood supply of the RV. MIs of the RV are frequently caused by RCA occlusions near their origin and the RV. Occlusions in the RCA often lead to MIs affecting the RV, and this is especially common in patients with a right-dominant heart who have a relative risk of 3.0 [6].

Electrocardiograms (ECGs) are crucial in diagnosing heart attacks by detecting specific changes in the heart's electrical activity, helping assess myocardial damage [7]. In 48% of cases, ECG changes are transient and resolve within 10 hours. A significant proportion of these studies focused on a single aspect of RV function, leaving numerous angiographic imaging comparison gaps. Traditional methods, such as measuring the area and volume of the RV, make it difficult to evaluate RV function. These issues arise as a result of the RV's complex structure and inability to define internal boundaries [8,9]. Innovative metrics that can measure the RV's longitudinal fibers contracting are highly desired. These fibers initiate the movement that draws a portion of the RV closer to its apex [10-12]. It is necessary to assess the function of the RV using "tissue Doppler" technology. The reliability of the myocardial performance index (MPI) is well established [13]. This MPI also assists researchers in their comprehension of recreational vehicle dynamics. The objective of the present research was to evaluate RV function in patients with acute inferior wall MI (IWMI) using echocardiography and correlate these findings with the degree of stenosis in the RCA observed in angiography.

## Materials and methods

Ethical approval and study design

The Rajendra Institute of Medical Sciences (RIMS), Ranchi, Jharkhand, Institutional Ethics Committee granted their approval for this study under memo number 250IEC, RIMS (date: June 14, 2021). Ethical guidelines were strictly followed in our study to ensure accuracy and reliability. Participants fully understood the study's goals before giving informed consent to participate.

Patient selection

The research was carried out at a teaching hospital in Ranchi, Jharkhand, between July 2021 and June 2022. It comprised 200 patients who were admitted to the coronary care unit within 24 hours of experiencing their first symptoms of acute inferior MI. Patients in the study were diagnosed with acute inferior MI based on a clinical evaluation, the patient's medical history, and ECG results, particularly an increase in the ST segment in the inferior leads (II, III, and aVF). A total of 140 patients were selected for the study after a rigorous selection procedure based on predetermined criteria including age, gender, medical history, and clinical symptoms.

Subject selection

Inclusion Criteria

Patients were included if they had an acute inferior MI, at least 30 minutes of chest pain, and an ECG with an ST-segment elevation of ≥0.2 mV in the inferior leads. An additional inclusion criterion was the presence of a right ventricle myocardial infarction (RVMI) associated with an inferior MI, which was defined by an ST-segment elevation of ≥0.1 mV in V4 through V6R lead. Participants had to have a favorable echocardiographic window for a precise evaluation. In addition, coronary angiography confirmation of significant stenosis in the RCA was an essential inclusion criterion.

Exclusion Criteria

The study did not include patients who were in critical condition or hemodynamically unstable and required immediate primary percutaneous coronary intervention (PCI). Additionally, individuals with significant lesions in the left anterior descending coronary artery, diffuse lesions in the RCA, or multivessel disease were excluded. Those with suboptimal echocardiographic views that hindered accurate assessments were also not considered for this study. Patients with a history of cor pulmonale, MI, atrial fibrillation, or significant valvular heart disease were likewise excluded from the study. We excluded patients with specific ECG patterns, bundle branch blocks, and conditions that can change ECG readings, such as pericarditis, myocarditis, and electrolyte imbalances. The presence or absence of these conditions was assessed through a combination of clinical evaluation, patient history, and diagnostic tests such as echocardiography, cardiac enzymes, and blood electrolyte levels. Finally, the study excluded patients whose culprit lesion was not identified during coronary angiography. The angiography results divided the patients who met these criteria into two groups: 58 with distal RCA stenosis (34 males and 24 females) and 82 with proximal stenosis (52 males and 30 females).

Clinical evaluation and laboratory tests

Clinical evaluations were performed precisely to identify signs of RV failure (RVF), such as hypotension and an increase in jugular venous pressure (JVP), particularly when there were clear lung fields present. Blood samples were taken for laboratory testing to determine the total blood count, lipid profile (total cholesterol, high-density lipoprotein, low-density lipoprotein, and triglycerides), and random blood sugar. Additionally, troponin T was measured, serving as an essential biomarker for assessing myocardial damage.

Conventional echocardiographic examination

Following initial stabilization and within the first 48 hours post-infarction, all patients underwent a comprehensive conventional echocardiographic evaluation using the General Electric (GE) HealthCare VividTM E95 echocardiographic system (GE HealthCare Technologies, Inc., Chicago, IL). This system had a flexible 1-5 MHz transducer and a tissue-specific Doppler imaging mode. Standard views recommended by the American Society of Echocardiography (ASE) were utilized during the examination. The peak tricuspid regurgitation (TR) jet velocity and the simplified Bernoulli equation were used to determine the RV systolic pressure (RVSP). This data was then combined with an estimate of right atrial (RA) based on the inferior vena cava diameter and respiratory variations. The RV's end-diastolic dimensions were measured using a four-chamber apical view centered on the RV. This view enabled the measurement of the RV's maximum diameter while also encompassing the heart's apex and crux.

RV function parameters

The measurement of tricuspid annular plane systolic excursion (TAPSE) was obtained by positioning an M-mode cursor across the tricuspid lateral annulus in an apical four-chamber view. The purpose was to quantify the longitudinal displacement of the annulus during peak systole. The pulsed-tissue Doppler imaging technique was utilized to evaluate cardiac dynamics. The images were captured through the apical four-chamber window, utilizing the tissue Doppler mode and focusing on the RV-free wall. At the end of expiration, the pulsed Doppler sample volume was placed at the tricuspid annulus of the basal RV free wall segment to minimize translational motion.

Comprehensive velocity profiles spanning the entire cardiac cycle were generated using the specific software, revealing distinct peaks and troughs. These included a significant positive peak systolic velocity (Sm), which indicated that the annulus was moving toward the apex during systole. In addition, negative early diastolic myocardial velocity (Em) measurements were recorded as the annulus ascended away from the apex. Following that, negative late diastolic myocardial velocity (Am) readings were recorded. The dynamic behavior of Sm, Em, and Am waves was effectively characterized by this analysis. Critical time intervals were meticulously measured to probe deeper into cardiac performance. The duration of Sm was calculated using the ejection time (ET). Isovolumic relaxation time (IVRT) data was obtained by calculating the time elapsed between the end of Sm and the start of Em. The duration of the interval between the end of Am and the beginning of Sm also provided insight into the isovolumic contraction time (IVCT).

The myocardial performance index (MPI), a key indicator of cardiac function, was calculated using the following formula: MPI = [(IVRT + IVCT) / ET].

Coronary angiography

Coronary angiography was performed within the initial week following MI to pinpoint the culprit lesion. This procedure involved the utilization of a Judkins catheter, following standardized techniques. The presence of total or subtotal occlusion of the coronary artery supplying the asynergic field was a crucial criterion for identifying the culprit lesion. Arteriography reveals that these characteristics frequently result from acute thrombosis or dissected plaque. This rigorous approach ensured the accurate definition of the culprit lesion. Based on the anatomical position of the RCA lesion in relation to the origin of the RV branch, the patients were divided into two different groups: group 1 included patients with the culprit lesion in close proximity to the RV branch of the RCA, and group 2 included patients with the culprit lesion far from the RV branch of the RCA.

Statistical analysis

STATA 11.0 (SPSS Inc., Chicago, IL) was used for descriptive statistics. To compare continuous variables, the Student t-test or Mann-Whitney test (depending on the data distribution) was utilized, whereas the Chi-square test was utilized for categorical variables. Multiple regression analyses determined the best RV function parameters for proximal RCA ischemia prediction (TAPSE, Sm, and MPI). A receiver operator characteristic curve modeled the most independent proximal RCA lesion predictors. Youden's equation found the best parameter cutoff for sensitivity and specificity. Echocardiographic features associated with angiographic RCA stenosis were recorded, analyzed, and concluded.

## Results

The study population was separated into two categories based on gender: males and females. Out of a total of 140 participants, 86 (61%) were males, while 54 (39%) were females (Table 1). Age distribution was further examined within the study population. Among those aged between 30 and 45 years, 20 (14%) participants were observed (Table 2). Of this group, 16 were males and four were females. Within the age range of 46-60 years, there were 70 (50%) participants. Out of these, 40 were males and 30 were females. Additionally, 50 (30%) patients were over 60. Among this older age group, 30 were males and 20 were females. Among the 86 male participants in the study, 52 (60.4%) were diagnosed with proximal RCA stenosis, while 34 (39.5%) presented with distal RCA stenosis (Table 3). Similarly, among the 54 female participants, 30 (55.5%) exhibited proximal RCA stenosis, and 24 (44.4%) displayed distal RCA stenosis.

**Table 1:** Distribution of gender within the study population

Gender	Male	Female	Total patients
Total (number (%))	86 (61)	54 (39)	140

**Table 2:** Distribution of age groups within the study population

Age groups (years)	Total patients (number (%))	Male (number (%))	Female (number (%))
30-45	20 (14)	16 (80)	4 (20)
46-60	70 (50)	40 (57)	30 (43)
>60	50 (36)	30 (60)	20 (40)

**Table 3:** Incidence of proximal and distal stenosis in the RCA across different age groups and genders RCA: right coronary artery

Age groups (years)	Proximal RCA stenosis (n = 41)		Distal RCA stenosis (n = 29)	
	Male (number (%))	Female (number (%))	Male (number (%))	Female (number (%))
30-45	10 (19)	3 (10)	6 (17.6)	1 (4.1)
46-60	18 (34.6)	14 (46.6)	22 (64.7)	16 (66.6)
>60	24 (46)	13 (43.3)	6 (17.6)	7 (29.2)
Total (number (%))	52 (60.4)	30 (55.5)	34 (39.5)	24 (44.4)

Delving further into the male population with proximal RCA stenosis (52 patients), the age distribution revealed that 10 (19%) individuals were within the age group of 30-45 years, and 18 (34.6%) patients fell between the ages of 46 and 60 years. The remaining 24 (46%) patients were aged above 60 years (Table 3). Among the male patients who had distal RCA stenosis (34 patients), the age breakdown was as follows: six (17.6%) patients belonged to the 30-45 years age group, 22 (64.7%) patients were in the 46-60 years age range, and six (17.6%) patients were older than 60 years. Shifting the focus to the female participants with proximal RCA stenosis (30 patients), their ages were distributed as follows: three (10%) individuals were between 30 and 45 years old, 14 (46.6%) patients were aged 46-60 years, and 13 (43.3%) patients were above 60 years old (Table 3). For female patients who presented with distal RCA stenosis (24 patients), the age distribution was detailed as follows: one (4.1%) individual was within the 30-45 years age bracket, 16 (66.5%) patients were aged 46-60 years, and seven (29.2%) patients were beyond 60 years of age (Table 3).

Risk factors present a comprehensive view of the prevalence of different risk factors among the study participants, categorized by their association with proximal and distal RCA stenosis (Table 4). In a study of 45 participants, 82.2% (37 individuals) with proximal RCA stenosis were diabetic, whereas only 17.8% (eight individuals) with distal RCA stenosis had diabetes. Hypertension was present in 66.2% (47 individuals) of the 71 participants with proximal RCA stenosis and 33.8% (24 individuals) of the 71 participants with distal RCA stenosis. In a separate group of 50 participants, 60% (30 individuals) of those with proximal RCA stenosis were diagnosed with dyslipidemia. In contrast, 40% (20 individuals) of those with distal RCA stenosis were diagnosed with the same condition. Of 42 participants with a smoking or tobacco use history, 47.6% (20 individuals) with proximal RCA stenosis were smokers or tobacco users, whereas 52.4% (22 individuals) with distal RCA stenosis were in the same category. There was no significant difference in epidemiological characteristics such as diabetes, hypertension, dyslipidemia, or smoking between the two study groups (Table 4).

**Table 4:** Relationship between risk factors and the occurrence of proximal and distal stenosis in the RCA RCA: right coronary artery

Risk factors	Total patients (number)	Proximal RCA stenosis (n = 41) (number (%))	Distal RCA stenosis (n = 29) (number (%))
Diabetes	45	37 (82.2)	8 (17.8)
Hypertension	71	47 (66.2)	24 (33.8)
Dyslipidemia	50	30 (60)	20 (40)
Smoking/tobacco addict	42	20 (47.6)	22 (52.4)

Among the patients with proximal RCA stenosis, systolic blood pressure (BP) and diastolic BP were 118 ± 18.6 and 79 ± 9.6 mmHg, respectively (Table 5), and the heart rate and body mass index (BMI) were 77 ± 15.4 bpm and 24.1 ± 2.8 kg/m², respectively. Among the patients with distal RCA stenosis, systolic BP and diastolic BP were 122 ± 16.8 and 81 ± 9.3 mmHg, respectively. The heart rate and BMI were 75 ± 12.2 bpm and 23.8 ± 2.6 kg/m², respectively. No significant difference was observed in systolic BP, diastolic BP, heart rate, or BMI between the two groups. Among the total of 140 patients, 44 had elevated JVP, out of which 40 (48.8%) had proximal RCA stenosis and four (6.7%) had distal RCA stenosis, which was clinically significant (Table 5). Among the 82 patients with proximal RCA stenosis, 28 (34%) were thrombolyzed, and among the 58 patients with distal RCA stenosis, 22 (38%) had received thrombolysis. There was no significant difference between the two groups (Table 5).

**Table 5:** Comparison of clinical parameters between proximal RCA stenosis and distal RCA stenosis groups in the present study *Note: The total patient (number) values pertain only to the thrombolytic therapy group. RCA: right coronary artery, BMI: body mass index, BP: blood pressure, JVP: jugular venous pressure, SD: standard deviation

Clinical parameters	Proximal RCA stenosis (mean ± SD)	Distal RCA stenosis (mean ± SD)
Systolic BP (mmHg)	118.0 ± 18.6	122.0 ± 16.8
Diastolic BP (mmHg)	79.0 ± 9.6	81.0 ± 9.3
Heart rate (bpm)	77.0 ± 15.4	75.0 ± 12.2
BMI (kg/m²)	24.1 ± 2.8	23.8 ± 2.6
Elevated JVP	40	4
Thrombolyzed (number (%))	28 (34)	22 (38)
Not thrombolyzed (number (%))	54 (66)	36 (62)
*Total patients (number)	82	58

This study examined echocardiographic variables to compare proximal and distal RCA stenosis patients. TAPSE stands out with a value of 12.7 ± 1.4 and 18.3 ± 2.4 in the proximal RCA stenosis group and the distal RCA stenosis group, respectively, with a significant difference (p ≤ 0.05) (Table 6). The right ventricular fractional area change (RVFAC) exhibits 32 ± 6.6 and 43 ± 6.4% in the proximal RCA stenosis and the distal RCA stenosis, respectively, signifying a noteworthy distinction (p ≤ 0.05) (Table 6).

**Table 6:** Comparative echocardiographic evaluation of proximal RCA stenosis and distal RCA stenosis groups in the current investigation RCA: right coronary artery, Am: late diastolic motion velocity, Em: early diastolic motion velocity, ET: ejection time, IVCT: isovolumic contraction time, IVRT: isovolumic relaxation time, LVEF: left ventricular ejection fraction, MPI: myocardial performance index, RVFAC: right ventricular fractional area change, Sm: systolic motion velocity, TAPSE: tricuspid annular plane systolic excursion, SD: standard deviation

	Proximal RCA stenosis (mean ± SD)	Distal RCA stenosis (mean ± SD)	p-value
TAPSE	12.7 ± 1.4	18.3 ± 2.4	≤0.05
RVFAC (%)	32 ± 6.6	43 ± 6.4	≤0.05
Sm (cm/s)	11.5 ± 2.6	15.4 ± 3.0	≤0.0001
Em (cm/s)	8.6 ± 2.8	9.4 ± 1.9	
Am (cm/s)	13.5 ± 3.8	14.8 ± 4.2	
Em/Am (ratio)	0.65 ± 0.18	0.69 ± 0.20	
IVRT (ms)	109 ± 18	85 ± 18	≤0.0001
IVCT (ms)	80 ± 14.4	74 ± 19	
ET (ms)	255 ± 20	288 ± 26	≤0.0001
MPI	0.75 ± 0.14	0.55 ± 0.16	≤0.0001
LVEF (%)	60 ± 3.8	61 ± 5.0	0.2

The Sm was significantly lower in the proximal RCA stenosis group (11.5 ± 2.6 cm/s) compared to the distal group (15.4 ± 3.0 cm/s), with a p-value of ≤0.0001 (Table 6). This suggests that proximal RCA stenosis patients have impaired systolic function. The proximal RCA stenosis group had an Em of 8.6 ± 2.8 cm/s, while the distal group had 9.4 ± 1.9 cm/s, and no significant difference was found in Em between groups (Table 6). The proximal RCA stenosis group had an Am of 13.5 ± 3.8 cm/s, while the distal group had 14.8 ± 4.2 cm/s, and Am also showed no significant variation between groups. The Em/Am ratio, which balances early and late diastolic velocities, was similar between groups. The proximal RCA stenosis group had a ratio of 0.65 ± 0.18, while the distal group had 0.69 ± 0.20 (Table 6).

The proximal RCA stenosis group had a significantly (p ≤ 0.0001) longer IVRT (109 ± 18 ms) than the distal group (85 ± 18 ms), indicating delayed relaxation. The IVCT showed no significant difference between groups. The proximal RCA stenosis group had an IVCT of 80 ± 14.4 ms, while the distal group had 74 ± 19 ms (Table 6). The ET was significantly (p ≤ 0.0001) shorter in the proximal RCA stenosis group (255 ± 20 ms) compared to the distal group (288 ± 26 ms), indicating altered systolic function. The MPI, a measure of cardiac function, was significantly (p ≤ 0.0001) higher in the proximal RCA stenosis group (0.75 ± 0.14) than in the distal group (0.55 ± 0.16). There was no significant difference (p = 0.2) in left ventricular ejection fraction (LVEF) between patients with proximal RCA stenosis (60 ± 3.8%) and those with distal RCA stenosis (61 ± 5%) (Table 6).

## Discussion

Accurate identification of the culprit artery is critical in the risk stratification and optimization of treatment approaches for patients afflicted by acute IWMI. Particularly, the mortality rate for acute IWMI cases accompanied by RV involvement attributable to proximal lesions within the RCA is notably elevated at 16%, in stark contrast to the 3.5% mortality rate observed in cases of isolated acute IWMI [14]. ECG assists in identifying the culprit artery; however, conventional ECG criteria have a low sensitivity for identifying the culprit artery in inferior ST-segment elevation myocardial infarction (STEMI) [15]. Echocardiography has become the standard RV evaluation method in routine clinical practice. Nevertheless, the complex RV geometry poses significant challenges to evaluating RV function [9]. In patients with RCA stenosis, few studies validate the utility of various ECG parameters of RV function. This study evaluated the validity of various RV function assessment parameters for predicting a proximal RCA lesion. The current study population comprised 86 (61%) males and 54 (39%) females, with a predominant representation of individuals above the age of 45 years. Notably, El Sebaie and El Khateeb [16] and Rajesh et al. [17] previously reported a closely similar trend where the majority of their respective study populations consisted of males, accounting for 79% and 83%, respectively, with an approximate age of 55 ± 12 years for both studies.

Our study reported that of the total study population of 140 patients, 58.5% had proximal RCA stenosis and 41.5% had distal RCA stenosis. This distribution contrasts with Rajesh et al. [17], who previously reported that 39% of patients had proximal RCA stenosis, and 61% had non-proximal RCA stenosis. Notably, the study by Rajesh et al. [17] included lesions involving both the RCA and left coronary artery (LCA) in cases of inferior wall MIs. El Sebaie and El Khateeb [16] examined 76 patients with inferior wall MIs and reported that 43 (56.5%) had proximal RCA stenosis, while 43.5% had distal RCA stenosis. Remarkably, our findings closely align with their results, reinforcing the consistency of outcomes across different studies. The variation in the distribution of proximal and distal RCA stenosis across study populations may be caused by factors such as patient demographics, regional cardiovascular trends, and diagnostic criteria used in various research settings.

Rajesh et al. [17] found that 43% of patients had diabetes and 50% had hypertension. Similarly, El Sebaie and El Khateeb [16] reported that 55% of participants had diabetes, 63% had hypertension, and 70% smoked. No statistically significant difference in risk factors was observed between the patients' proximal RCA and non-proximal RCA lesion groups in these two studies. The distribution of population risk factors in our study aligns with the findings of the studies mentioned above. In the present investigation, we did not observe any statistically significant disparities in systolic and diastolic BP, BMI, or heart rate among patients afflicted with proximal and distal RCA stenosis. Consistent with El Sebaie and El Khateeb [16], our study revealed no significant differences in systolic and diastolic BP or heart rate between patients with stenosis in the proximal and distal regions of the RCA. It is worth mentioning that individuals with proximal RCA stenosis exhibited a considerably higher occurrence of RVMI compared to those without this condition. However, the findings of Rajesh et al. [17] indicate a significant distinction in systolic and diastolic BP levels between individuals diagnosed with IWMI and proximal RCA lesions and those with non-proximal RCA and LCA lesions.

In the current study, a 10-fold increase in elevated JVP was observed in proximal RCA stenosis compared to distal RCA stenosis, which was clinically significant. This finding suggests a potential association between proximal RCA stenosis and increased JVP, which may reflect RV function and hemodynamics variations. Further investigation into this observation's underlying mechanisms and clinical implications is required to better comprehend its implications for patient management and risk evaluation. There was no significant difference in thrombolysis rates between the proximal and distal RCA stenosis groups in the current study, implying that the decision to administer thrombolytic therapy was unaffected by the location of the RCA stenosis. The lack of a significant difference in thrombolysis rates between patients with proximal and distal RCA stenosis may reflect healthcare professionals' clinical judgment to provide appropriate treatment regardless of the anatomical location of the stenosis. However, the decision to use thrombolytic therapy in any STEMI presentation, irrespective of location, should be based on clinical assessment and guideline adherence, especially when PCI is delayed. It is important to remember that the decision to undergo thrombolysis was most likely influenced by several factors, including the severity of symptoms, the extent of ischemia, the patient's overall condition, and the length of time since symptoms first appeared.

In our study, TAPSE was found to be lower in patients with proximal RCA stenosis than those with distal RCA stenosis, which was statistically significant (p ≤ 0.05). Previous research has found a strong link between TAPSE and ECG evidence of RVMI [18]. Furthermore, TAPSE has been identified as an independent predictor of mortality in cases of inferior wall MI. In studies, TAPSE has also been linked to radionuclide-derived ejection fraction (EF) [19]. According to ASE guidelines, a TAPSE value of less than 16 indicates RV systolic dysfunction [20]. However, El Sebaie and El Khateeb [16] did not observe a statistically significant disparity in TAPSE between the two cohorts under investigation. This outcome was attributed to the inherent constraint of TAPSE in accurately assessing the overall performance of a multifaceted structure through single-segment analysis [20]. Furthermore, the TAPSE cutoff value exhibits a notable degree of specificity but a relatively lower level of sensitivity when differentiating between patients with abnormal conditions and those considered normal. Rajesh et al. [17] observed a significant decrease in TAPSE in patients with proximal RCA lesions. Our findings support using TAPSE to assess RV function, particularly in proximal RCA stenosis; however, TAPSE's single-segment analysis and cutoff value must be understood. These factors emphasize the need for comprehensive TAPSE interpretation in clinical practice.

Our 2D echo analysis showed that patients with proximal and distal RCA stenosis had statistically significant (p < 0.05) differences in RVFAC. RVFAC, an indicator of RV systolic function, was lower in patients with proximal RCA stenosis. This highlights its potential as an indicator of myocardial impact localization. FAC, which measures the overall systolic function of the RV, correlates with cardiac magnetic resonance imaging (MRI)-measured RV ejection fraction [21]. In addition, its prognostic significance in patients with MI has been established [22], enhancing its clinical significance. Rajesh et al. [17] reported that patients with proximal RCA stenosis had a lower RVFAC (32 ± 5.2%) than those without proximal RCA stenosis (44 ± 5.2%). Similar to their research, our study highlights the diagnostic potential of RVFAC for identifying proximal RCA lesions. This knowledge can assist in enhancing risk stratification and treatment planning for patients who have experienced an acute coronary event.

We found a statistically significant difference (p < 0.0001) in Sm between patients with proximal RCA stenosis and distal RCA lesions. Ozdemir et al. [23] supported these findings by highlighting the close relationship between Sm and patients with proximal RCA involvement in inferior wall MI. Moreover, Dokainish et al. [24] highlighted Sm's potential as a predictor of RVMI. Wang et al. [25] discovered a correlation between tricuspid Sm and RV ejection fraction as measured by cardiac magnetic resonance imaging. The lack of significant differences in the parameters Em, Am, and Em/Am ratio between the two groups in our study raises the possibility that certain aspects of cardiac function related to these measurements may be similar. The Em/Am ratio offers information about diastolic function because Em and Am stand for early and late diastolic velocities of mitral annular motion, respectively.

Early filling immediately tracks the end of the systolic motion in a normal RV with intact contractility and normal loading conditions, and the RV IVRT needs to be more brief and present. Consequently, a detectable IVRT indicates elevated RV end-systolic pressure [26]. In our study, patients with proximal RCA exhibited significantly longer RV IVRT and shorter ET. This is because proximal RCA patients have a significantly higher incidence of RVMI. Furthermore, our study revealed that the prolongation of IVCT and the decrease in ET were the main factors that contributed to a significantly elevated MPI in patients with stenosis in the RCA, with statistical significance at p < 0.0001. This indicates that patients with proximal RCA stenosis have a higher level of cardiac dysfunction. The MPI has been established as a reliable indicator for identifying proximal RCA lesions because it provides a comprehensive measurement that includes both the systolic and diastolic phases of the cardiac cycle [27]. The research by Møller et al. [28], which showed higher MPI values in both the left and right ventricles during the hyperacute phase of MI compared to a control group, is consistent with the findings presented in this study. Our study's results show that the two groups with proximal and distal RCA stenosis did not significantly differ (p = 0.2) in their LVEF. Accordingly, the anatomical location of the stenosis along the right coronary artery may not significantly impact the LVEF.

Limitations

Several limitations were acknowledged when interpreting the study's findings. An echocardiographic evaluation should precede any reperfusion strategy to account for the possible recovery of RV function. Ethical considerations precluded delaying reperfusion to perform an echocardiographic evaluation. In addition, there is a possibility that later coronary angiograms missed cases of spontaneously reopened proximal RCA occlusions. In addition, it is important to note that a proximal RCA lesion does not necessarily indicate RV myocardial involvement, even though it is recognized as an independent prognostic indicator in acute inferior wall IWMI cases.

## Conclusions

In summary, the comprehensive echocardiographic evaluation of diverse parameters relating to RV function in the context of acute inferior wall STEMI has revealed substantial differences between groups characterized by proximal and distal RCA lesions. Among 140 participants, mostly males (61%) aged above 45 years, key findings emerged. The utility of easily accessible measures such as tissue Doppler systolic annular velocity, MPI, and TAPSE has been underscored in predicting the proximal RCA as the infarct-related artery. It is noteworthy that all patients diagnosed with proximal RCA stenosis exhibited RV dysfunction upon echocardiographic analysis. Importantly, the study's findings also emphasize a high incidence of RVMI in patients with proximal RCA stenosis, reinforcing the clinical significance of these observations.
